# Development and validation of a deep learning-based protein electrophoresis classification algorithm

**DOI:** 10.1371/journal.pone.0273284

**Published:** 2022-08-24

**Authors:** Nuri Lee, Seri Jeong, Kibum Jeon, Wonkeun Song, Min-Jeong Park

**Affiliations:** 1 Department of Laboratory Medicine, Kangnam Sacred Heart Hospital, Hallym University College of Medicine, Seoul, South Korea; 2 Department of Laboratory Medicine, Hangang Sacred Heart Hospital, Hallym University College of Medicine, Seoul, South Korea; United International University, BANGLADESH

## Abstract

**Background:**

Protein electrophoresis (PEP) is an important tool in supporting the analytical characterization of protein status in diseases related to monoclonal components, inflammation, and antibody deficiency. Here, we developed a deep learning-based PEP classification algorithm to supplement the labor-intensive PEP interpretation and enhance inter-observer reliability.

**Methods:**

A total of 2,578 gel images and densitogram PEP images from January 2018 to July 2019 were split into training (80%), validation (10%), and test (10.0%) sets. The PEP images were assessed based on six major findings (acute-phase protein, monoclonal gammopathy, polyclonal gammopathy, hypoproteinemia, nephrotic syndrome, and normal). The images underwent processing, including color-to-grayscale and histogram equalization, and were input into neural networks.

**Results:**

Using densitogram PEP images, the area under the receiver operating characteristic curve (AUROC) for each diagnosis ranged from 0.873 to 0.989, and the accuracy for classifying all the findings ranged from 85.2% to 96.9%. For gel images, the AUROC ranged from 0.763 to 0.965, and the accuracy ranged from 82.0% to 94.5%.

**Conclusions:**

The deep learning algorithm demonstrated good performance in classifying PEP images. It is expected to be useful as an auxiliary tool for screening the results and helpful in environments where specialists are scarce.

## 1. Introduction

Protein electrophoresis (PEP) indirectly identifies characteristic patterns and increases or decreases the concentration of individual proteins by separating several individual proteins, including albumin, present in high serum concentrations. In PEP, serum proteins are separated by electrophoresis into five or six major fractions: albumin, alpha 1, alpha 2, beta 1, beta 2, and gamma globulin [1, 2]. PEP has been utilized in clinical practice, as the change in concentration and pattern of these proteins in patients is related to various diseases [3, 4]. It is the most widely used method for detecting monoclonal bands in gamma globulin, which is an indicator of uncontrolled growth and division of malignant plasma cells in the form of monoclonal immunoglobulins in patients with multiple myeloma. PEP has been established as an essential test for the diagnosis and follow-up of multiple myeloma [5, 6]. In addition, presenting a specific pattern, it can be implemented for the diagnosis of diverse diseases, such as nephrotic syndrome, liver cirrhosis, protein loss bowel disease, and hypogammaglobulinemia [4, 7, 8]. However, as PEP is interpreted based on visual reading, not only relative quantitative values but also specific shapes of curves, an expert’s proficiency in reading the gel or densitogram graph greatly influences the accuracy of the examination. Interpretation of PEP curves requires experienced operators to understand the overall clinical conditions of patients because they can be affected by various pathological conditions, as well as endogenous and exogenous potential interfering factors [9–11]. In particular, follow-up examinations of patients undergoing treatment or interpretation of patients with multiple underlying diseases require considerable caution in PEP reading [12–14]. In most laboratories, the reading step by the experts is a major factor in delaying the reporting of results, and the need for an auxiliary tool for interpretation has been raised [14–16]. Further, morphologic evaluation has the limitation of inter-pathologist reliability, which incurs difficulty in standardizing the interpretation criteria [17].

Recently, artificial intelligence (AI) technology has been rapidly progressing and widely adopted in various medical fields [18–21]. These technologies are not only used for simple image segmentation and classification, but also make it possible to convert various non-image data into well-organized image-form through a convolution neural network (CNN) [20, 21]. PEP also has the potential for various clinical applications of AI algorithms using existing accumulated data, and a few deep learning (DL)-related PEP analyses have been conducted in recent years. To date, several algorithms have been developed for detecting both normal and pathogenic patterns of PEP capillary images. However, the specificity of the developed algorithms is not high, and only limited data were employed in their development [15, 16]; this increases the need for additional research with various DL algorithms. Thus, the possibility of DL-based classification in patients with kidney, liver, and neurological diseases should be evaluated by developing a DL algorithm with a large-scale PEP image database.

In this study, we developed and evaluated a DL-based PEP classification algorithm for patterns with monoclonal gammopathy, acute-phase proteins, hypoproteinemia, nephrotic syndrome, polyclonal gammopathy, and normal. Herein, we report on its development and evaluation, present applications of this algorithm in actual clinical practice, and suggest future tasks for the development of DL algorithms related to PEP interpretation.

## 2. Materials and methods

### 2.1. Dataset

**Fig 1:**
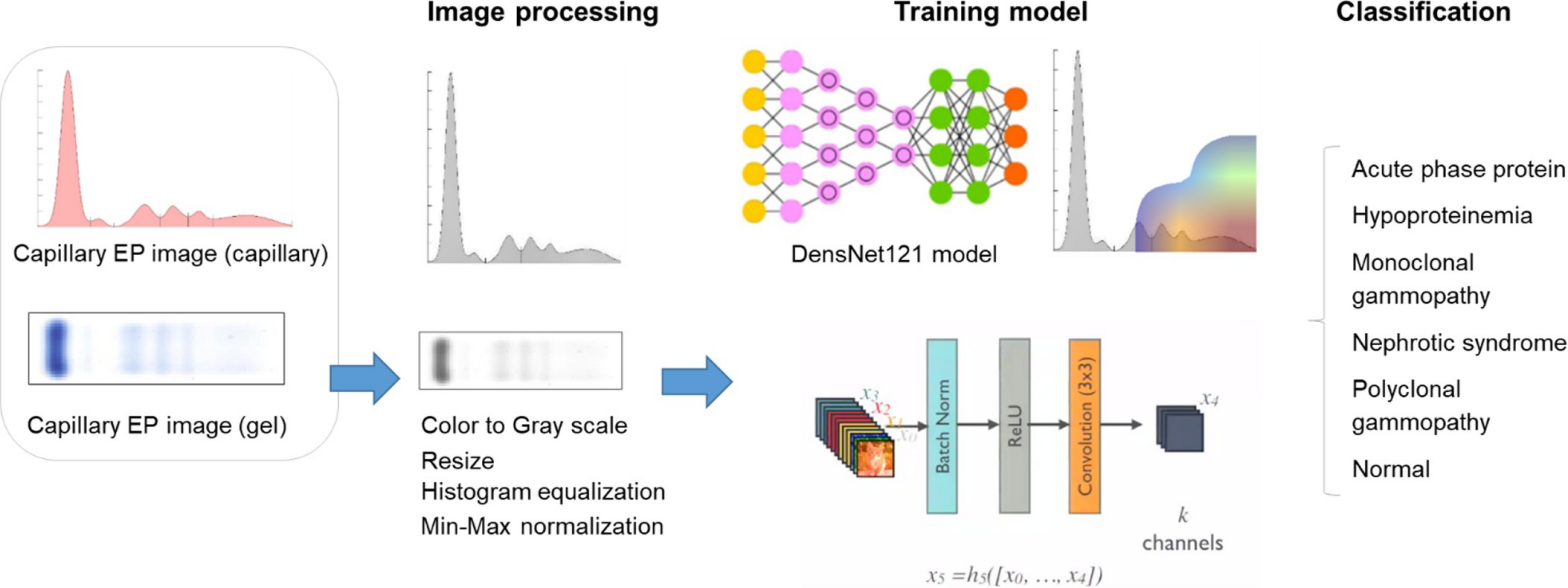
Dataset preparation and proposed framework.

An overview of the dataset preparation and proposed framework is presented in Fig 1. The PEP images were obtained at Kangnam Sacred Heart Hospital, from the assay results of a SPIFE 3000 electrophoresis system (Helena Laboratories, Beaumont, TX, USA). Both the scanned PEP gel images and converted densitogram images, which were collected from January 2018 to July 2019, were used for the analysis. PEP gel and densitogram images were collected and paired from 1076 patients, and median 2.0 (95% CI = 2.0–3.0) gel or densitogram images of different dates were collected from 88 patients. For each patient, demographic data, including age, sex, total protein, and albumin at the date of the collection, were investigated. This study was approved by the institutional review board at Kangnam Sacred Heart Hospital (institutional review board identifier: HKS 2020-03-022) and was conducted per the tenets of the Declaration of Helsinki. The need for informed consent for this study was waived, as the anonymity of personal information was maintained throughout the study.

Among the results of PEP, patients who had reported six major findings (i.e., acute-phase protein, monoclonal gammopathy, polyclonal gammopathy, hypoproteinemia, nephrotic syndrome, and normal) were included in the study. The PEP gel and densitogram sample images of each of the six major findings were provided in supporting information (S1 Fig). All images were retrospectively reviewed independently by following the published standard guidelines [4, 22] by two pathologists with 5 and 22 years of laboratory medicine experience. Every image was reviewed by both pathologists, and any disagreement between them was resolved by consensus. The reference standard for the diagnosis of monoclonal gammopathy was based on immunofixation electrophoresis (IFE) results, among images with characteristic sharp patterns in the beta or gamma region. Polyclonal gammopathy was designated as an image showing swell-like gamma elevation due to an increase in the gamma region. Images with increased alpha fraction, a normal to a mild increase in total protein, and a normal to a mild decrease in albumin on the chemistry analyzer were labeled as acute-phase protein. In contrast, images showing a marked increase in the alpha-2 region but decreased total protein and albumin fractions were labeled as nephrotic syndrome. For hypoproteinemia, the amount of measured total protein was lower than the reference range, and images with reduced albumin fraction were included. In the case of normal, each region was designated as being within the reference range. The increase or decrease of each protein region was determined according to the reference range established in the laboratory.

After completion of the annotation, all dataset images were randomly split into training (80%), validation (10%), and test (10.0%) sets. The PEP images underwent color-to-grayscale conversion, resizing to 256 ⅹ 256 pixels, and image histogram equalization. After the images were processed, they were input into neural networks.

### 2.2. Development and evaluation of algorithms and statistical analyses

The performance of the DL algorithms was evaluated using in-house test datasets. The area under the receiver operating characteristic curve (AUC) was computed for each diagnosis. The sensitivity, specificity, positive predictive value (PPV), and negative predictive value (NPV) that yielded the highest performance were estimated. The numbers on the curve represent the degree of accuracy as follows: no discrimination (AUC < 0.5), acceptable (0.5 ≤ AUC < 0.7), excellent (0.7 ≤ AUC < 0.9), and outstanding (0.9 ≤ AUC) [23]. We used the metrics modules in DEEP:PHI (medical AI software; DEEPNOID, Seoul, Republic of Korea), which is an open platform that assists DL model research. Further, statistical analyses were performed using the DEEP:PHI platform. We used the DenseNET-121 architecture, a well-known object detection DL framework, to perform a per-image diagnosis of the PEP results [24]. In addition, we utilized other DL algorithms, such as VGG19 [25], InceptionV3 [26], and Xception [27], to compare the performance among various algorithms. Adaptive Moment Estimation (Adam) optimizer was utilized for hyperparameter settings with a learning rate of 0.0001, selected by trial-and-error approach. The gradient decay factor was set to 1.0. The batch size value was 16, and the number of epochs equals 30. The Gradient-weighted Class Activation Mapping (Grad-CAM) technique was used for the interpretation and evaluation of DL outputs [28].

## 3. Results

### 3.1. Demographic and clinical characteristics

We collected annotations for 2578 images during the study period. Table 1 lists the number of images for each of the six findings in our in-house dataset. We utilized 1033 densitogram EP images (80.0%) for training, 128 images (10.0%) for validation, and 128 images (10.0%) for testing. The same numbers of gel EP images (1033 for training, 128 for validation, and 128 for testing) were collected for evaluation of the gel EP dataset. The train, validation, and test sets were split independently at the gel or densitogram images level. The six-tiered diagnosis included acute-phase protein (N = 148), hypoproteinemia (N = 498), monoclonal gammopathy (N = 528), nephrotic syndrome (N = 330), polyclonal gammopathy (N = 288), and normal patterns (N = 586). There were no statistically significant differences in median age, gender, total protein, and albumin among training, validation, and test sets in both the densitogram and gel image data sets.

**Table 1:** Demographic statistics and diagnostic classification of protein electrophoresis datasets.

	Densitogram PEP (N = 1289)		Gel PEP (N = 1289)	
	Training and Validation	Test	Training and Validation	Test
Age (year)	67.0 (55.0–77.0)	66.0 (51.0–75.5)	67.0 (55.0–76.3)	64.0 (52.0–78.0)
Sex (Male:Female)	604 : 557	68 : 60	610 : 551	62 : 66
Total protein (mg/dL)	6.4 (5.6–7.2)	6.4 (5.6–7.2)	6.2 (5.5–7.0)	6.4 (5.6 0 7.3)
Albumin (mg/dL)	3.4 (2.7–3.9)	3.3 (2.7–3.8)	3.3 (2.7–3.8)	3.2 (2.7–3.8)
Total no. of EP images	1161	128	1161	128
Diagnosis				
Acute-phase protein	69 (5.9%)	5 (3.9%)	65 (5.6%)	9 (7.0%)
Hypoproteinemia	223 (19.2%)	26 (20.3%)	224 (19.3%)	25 (19.5%)
Monoclonal gammopathy	235 (20.2%)	29 (22.7%)	240 (20.7%)	24 (18.8%)
Nephrotic syndrome	149 (12.8%)	16 (12.5%)	144 (12.4%)	21 (16.4%)
Polyclonal gammopathy	222 (19.1%)	22 (17.2%)	224 (19.3%)	20 (15.6%)
Normal	263 (22.7%)	30 (23.4%)	264 (22.7%)	29 (22.7%)

Values are presented as median (interquartile range).

### 3.2. Diagnostic performance of the deep learning model for the six-tiered diagnosis

The DenseNET-121 architecture showed better AUC for most of the PEP densitogram patterns (acute phase protein, hypoproteinemia, monoclonal gammopathy, nephrotic syndrome), when compared with the other algorithms; specifically, InceptionV3, and Xception (Table 2). However, in the case of gel PEP images, three algorithms exhibited similar performance with each other, and no architecture consistently outperformed the others, among them.

**Table 2:** Comparisons of the area under the receiver operating characteristic curve (AUC) of Inception V3, Xception, and DenseNET-121 to identify patterns of protein electrophoresis images.

	Densitogram EP image			Gel EP image		
	Inception V3	Xception	DenseNET-121	Inception V3	Xception	DenseNET-121
Acute phase protein	0.647	0.826	0.873	0.767	0.665	0.763
Hypoproteinemia	0.833	0.856	0.891	0.888	0.879	0.863
Monoclonal gammopathy	0.970	0.952	0.979	0.920	0.920	0.897
Nephrotic syndrome	0.936	0.963	0.967	0.894	0.910	0.919
Polyclonal gammopathy	0.993	0.991	0.989	0.942	0.977	0.965
Normal	0.942	0.933	0.927	0.933	0.934	0.929

**Fig 2:**
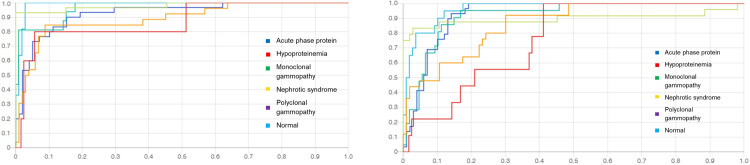
ROC curves for classification of diagnosis from PEP images. (A) densitogram EP images and (B) gel EP images.

The AUC, sensitivity, specificity, accuracy, PPV, and NPV for the six findings with DenseNET-121 were presented in Table 3. In the case of densitogram EP images, the AUC for monoclonal gammopathy was 0.979, with a sensitivity of 86.2% and specificity of 100%. The sensitivities for polyclonal gammopathy, hypoproteinemia, acute-phase protein, nephrotic syndrome, and normal were 81.8%, 84.6%, 60.0%, 68.7%, and 66.7%, respectively. Specificity was much higher, at 99.1% for nephrotic syndrome, 98.1% for polyclonal gammopathy, 95.1% for acute-phase protein, 94.9% for normal pattern, and 85.3% for hypoproteinemia. Fig 2 presents the receiver operating characteristic (ROC) curve for each diagnosis. The figure also shows dependable results for polyclonal gammopathy (0.989), followed by nephrotic syndrome (0.967), monoclonal gammopathy (0.979), normal pattern (0.927), hypoproteinemia (0.891), and acute-phase protein (0.873) (Table 3 and Fig 2A). When gel EP images were applied, each evaluation parameter showed a decreased performance. The sensitivity for diagnosis ranged from 22.2% to 80.0%, and the specificity ranged from 87.9% to 98.1%. The AUC for the gel EP images also slightly decreased, with the highest value for polyclonal gammopathy (0.965) and the lowest for acute-phase protein (0.763) (Table 2 and Fig 2B).

**Table 3:** Summary of performance, including AUROC, for each finding in the database.

(A) Densitogram EP image						
	Metric					
	Sensitivity	Specificity	AUROC	Accuracy	PPV	NPV
Acute phase protein	0.600	0.951	0.873	0.937	0.333	0.983
Hypoproteinemia	0.846	0.853	0.891	0.852	0.595	0.956
Monoclonal gammopathy	0.862	1.000	0.979	0.969	1.000	0.961
Nephrotic syndrome	0.687	0.991	0.967	0.953	0.917	0.957
Polyclonal gammopathy	0.818	0.981	0.989	0.953	0.900	0.963
Normal	0.667	0.949	0.927	0.883	0.800	0.903
(B) Gel EP image						
	Metric					
	Sensitivity	Specificity	AUROC	Accuracy	PPV	NPV
Acute phase protein	0.222	0.882	0.763	0.836	0.125	0.938
Hypoproteinemia	0.520	0.893	0.863	0.820	0.542	0.885
Monoclonal gammopathy	0.792	0.981	0.897	0.945	0.905	0.953
Nephrotic syndrome	0.238	0.972	0.919	0.852	0.625	0.867
Polyclonal gammopathy	0.800	0.917	0.965	0.898	0.640	0.961
Normal	0.759	0.879	0.929	0.852	0.647	0.926

### 3.3. True and false-positive prediction by the algorithm

**Fig 3:**
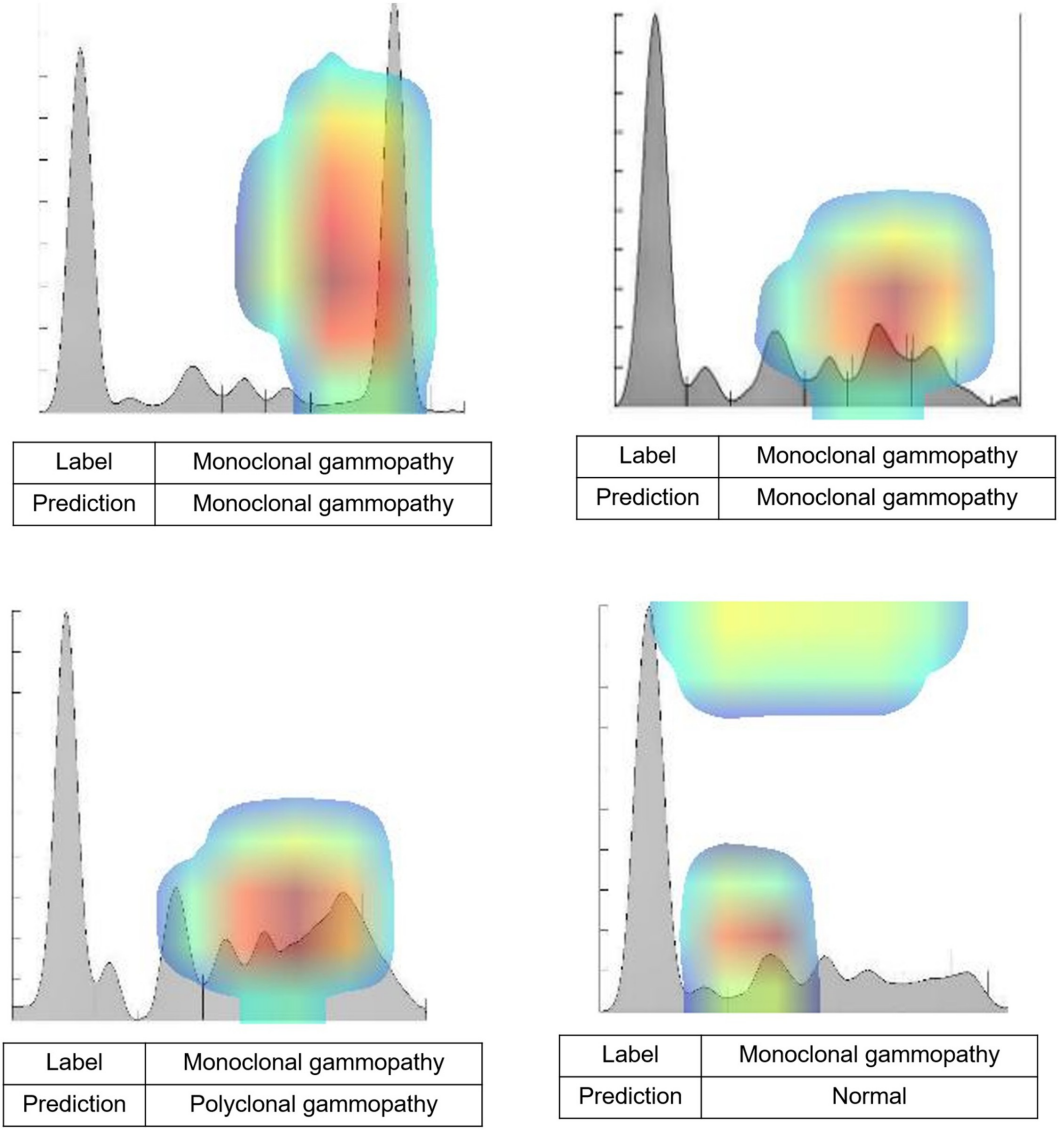
Representative true and false-positive case images results from Gradient-weighted Class Activation Mapping (Grad-CAM), obtained using DenseNET-121 classification model. (A) and (B) show true positive cases with a definite monoclonal peak and small monoclonal peak, respectively. Patients A and B showed 4.3g/dL and 1.1g/dL M-peaks (IgG, kappa type pattern with immunofixation assay). Monoclonal gammopathy cases were incorrectly predicted as polyclonal gammopathy (C) and normal (D). Patient C showed a 0.7 g/dL M-peak (IgG, lambda), and patient D showed a 0.6g/dL M-peak (bi-clonal band with IgG, kappa).

Table 4 lists the true and false-positive results according to the algorithm. In the case of monoclonal gammopathy for densitogram images, 25 out of 29 tested images showed correct results (Table 4A). Fig 3A and 3B show examples of true positive results for monoclonal gammopathy. It was possible to achieve the correct results for small peaks as well as definite peaks. False-positive results were given for two hypoproteinemias, one polyclonal gammopathy (Fig 3C), and one normal (Fig 3D) image. In the case of polyclonal gammopathy, four images showed false-positive results, and two each were predicted as hypoproteinemia and normal images. Regarding acute-phase proteins, there were two false-positive results, which were predicted differently for nephrotic syndrome and hypoproteinemia. Meanwhile, the nephrotic syndrome was difficult to predict when distinguishing it from acute-phase proteins or hypoproteinemia. Table 4B provides the results of the gel images. There were more incorrect predictions than for the densitogram images; in particular, the numbers of incorrect predictions for normal (5 vs. 12) and polyclonal gammopathy (2 vs. 9) were higher than for the densitogram images. The examples of true- and false-positive gel PEP images were presented in supporting information (S2 Fig).

**Table 4:** Confusion Matrix for disease diagnosis from the PEP dataset.

(A) Densitogram EP image							
Label	Prediction						
	APR	Hypoproteinemia	Monoclonal gammopathy	Nephrotic syndrome	Polyclonal gammopathy	Normal	Total
APR	3	1	0	1	0	0	5
Hypoproteinemia	2	22	0	0	0	2	26
Monoclonal gammopathy	0	2	25	0	1	1	29
Nephrotic syndrome	2	3	0	11	0	0	16
Polyclonal gammophathy	0	2	0	0	18	2	22
Normal	2	7	0	0	1	20	30
Total	9	37	25	12	20	25	128
(B) Gel EP							
Label	Prediction						
	APR	Hypoproteinemia	Monoclonal gammopathy	Nephrotic syndrome	Polyclonal gammopathy	Normal	Total
APR	2	3	0	1	1	2	9
Hypoproteinemia	2	13	0	1	4	5	25
Monoclonal gammopathy	1	1	19	1	1	1	24
Nephrotic syndrome	9	4	1	5	1	1	21
Polyclonal gammophathy	0	0	1	0	16	3	20
Normal	2	3	0	0	2	22	29
Total	16	24	21	8	25	34	128

## 4. Discussion

In this paper, we reported the development and validation of a DL-based PEP classification algorithm for the identification of various patterns. We developed an algorithm to detect monoclonal gammopathy and demonstrated its performance with 86.2% sensitivity and 100% specificity. This algorithm showed favorable performance when applied to the diagnosis of nephrotic syndrome, polyclonal gammopathy, and normal patterns, among others, with AUC values of over 0.9.

To the best of our knowledge, DL-based studies for the classification of various PEP patterns have not been sufficiently performed [1, 15, 16, 29, 30]. Only a few studies have been conducted, but there have been limitations for actual clinical application. Ognibene et al. were the first to apply an artificial neural network-based algorithm to PEP, but it only discriminated the PEP images by “normal vs. pathological” patterns, and their definition of pathological image is unclear [16]. Altinier et al. also focused only on the anomaly of each fraction rather than on the comprehensive interpretation and diagnosis of PEP [1]. More recently, a PEP analysis DL algorithm using large-scale images was developed by Floris et al. [30]. Our study differs from theirs in that our classification was implemented through image training using both expert reading and test results (IFE, protein level) without separation of fractions, and a more detailed clinical diagnosis was applied. Further, in the detection of M-spike, the accuracy of our algorithm was slightly higher than that of Floris et al. (91.2% vs. 96.9%).

In the present study, large amounts of various PEP image patterns with annotation by specialists were applied, and a DL-based classification algorithm that directly and specifically interprets PEP images was developed. In addition, improved performance was also demonstrated based on the various DL algorithm techniques, which were more advanced than in most previous studies. We found the most optimal algorithm by comparing the performance of various recently developed algorithms. Only a few studies have compared the performance of various DL algorithms for application to actual clinical practice, and comparative evaluation in laboratory medicine remains insufficient [31]. Currently, various DL algorithms have been rapidly progressed and developed, each with different characteristics. VGG19 is a VGGNET neural network model with 19 convolution layers. It is characterized by using a relatively small 3x3 or 1x1 kernel to deepen the network [25]. InceptionV3 is an evolution of the previous GoogleLeNet. It uses filters of several sizes concurrently and also uses a smaller size filter to reduce the number of dimensions; it has the advantage of increasing the computation efficiency [26]. Xception has the characteristic that it can learn at the same time by separating channel information and spatial information by applying a depth-wise separable convolution method [27]. DenseNET-121 is a neural network structure in which the dense connectivity method is applied to CNN DL training, and it has the advantage of alleviating the gradient vanishing problem. In addition, because the number of parameters is reduced compared to the depth of the network, efficient computation is possible and improved performance can be achieved even with a small dataset [24]. The DenseNET-121 architecture used in this study has shown favorable performance in many image analysis studies [32, 33], and it demonstrated the best accuracy among various DL algorithms. Furthermore, various novel approaches that allow high-throughput biological data have continuously appeared [20, 21]. These approaches enable the conversion of nonimage data into a form that is compatible with CNN architectures.

In this study, the Grad-CAM heatmap-generating technique was applied for CNN interpretation [28]. Utilizing this technique, the region of interest on a PEP image was highlighted, so that the significant region of the image for prediction could be focused on, aiding the interpretation of the image. When investigating the diagnostic failures of this study with Grad-CAM, it was possible to infer several reasons for false positives or false negatives. In the case of monoclonal gammopathy, when polyclonal gammopathy was accompanied, or when the monoclonal peak was atypical and very tiny, the prediction was limited even though the gamma region was included in the significant region by Grad-CAM. In the case of polyclonal gammopathy, when the area of the gamma region was small, and the alpha region was relatively large, the significant region was regarded as the alpha to beta region, and it was incorrectly analyzed as normal or hypoproteinemia. In the case of the distinction between hypoproteinemia and normal or nephrotic syndrome and acute-phase protein, quantitative values through a chemical analyzer were considered in the reading; thus, it was difficult to distinguish them if they did not present a typical peak.

In addition, in this study, the performances of algorithms derived from densitogram images and gel images for PEP were compared. The gel EP images were found to have lower overall performance than densitogram images. Densitogram images are more intuitive than gel images and are easy to read with visual assessment, and the evaluation assessed by the DL algorithm also showed similar results. Densitogram PEP images (or capillary images) will be preferred over gel EP images in future DL applicable studies using PEP images. This study is expected to be used as a reference to determine the appropriate algorithm or type of image for various DL studies based on PEP images in the future.

PEP is an essential tool for the diagnosis of monoclonal gammopathy [5, 6]. As the incidence of multiple myeloma disease gradually increases, the number of assays has expanded significantly [34]. The interpretation and reporting of PEP results are currently performed by specialists in laboratory medicine, but a considerable amount of time is required for proper reading by specialists. In addition, the detection of monoclonal gammopathy by PEP is subjective and false-positive cases of monoclonal gammopathy by the EP method have occasionally occurred [14]. Furthermore, there are no standardized clinical practice guidelines for the interpretation of PEP [35]. In this study, all monoclonal gammopathy specimens confirmed by the IFE study were trained, and both the typical peak and atypical peak, including a small peak or peak outside the gamma region, could be detected with high accuracy. In the case of monoclonal proteins, not only detection of monoclonal components but also standardization of the M protein measurement process and establishment of clinical practice guidelines are important issues [35]. DL-based classification algorithms are helpful for the standardization of M component detection and quantification as they provide a more objective interpretation. The proposed algorithm is expected to be useful not only as an auxiliary tool to aid institutions that lack specialized manpower but also to decrease the variability of morphologic assessment that constantly helps discrimination even for difficult-to-distinguish peaks.

Although PEP has been suggested to apply to various diseases in several previous reports, it has not been actively used in clinical practice because of limitations such as difficulty in test execution and the time required for interpretation of the test [4, 17]. In this study, hypoproteinemia and acute-phase protein patterns showed AUC values higher than 0.8, and nephrotic syndrome, polyclonal gammopathy, and normal patterns showed AUC values exceeding 0.9. Although the PEP image pattern included in this study exhibited a relatively low sensitivity, it has high specificity and NPV, suggesting the possibility of being useful as an auxiliary tool in the exclusion of diseases in the screening step.

This study had a limitation that only internal data were used, and there was a restriction on the expansion of the evaluation results. Because the densitogram image appears as a regular pattern regardless of equipment or location, it is considered that the difference between institutions is not large, and it has been reported that the performance difference between internal data and external data was not significant in similar studies conducted previously [30]. However, to maximize reliability with limited images, efforts such as reducing the label errors through review, reconfirming duplication and/or omitted values, and reducing image noise were made. Through further studies, image augmentation and generalization through external data are necessary. In addition, various other state-of-art algorithms, including approaches that implement a CNN to nonimage, could be utilized for PEP interpretation. Since PEP is mostly read with reference to various test results (albumin, hemolysis, immunoglobulin, creatinine, inflammatory markers) reported as text, the development of a reading algorithm model that integrates clinical data is essential for actual clinical application. In follow-up studies, we intend to supplement the algorithm by including more images with external data and integrating related laboratory data with images.

## 5. Conclusions

In this paper, we reported the development of a DL-based interpretation algorithm using PEP images. We obtained acceptable to excellent performance evaluation results, with an AUC of 0.873–0.989 and an accuracy of 0.852–0.969 for various patterns. DL-based reading may enable a reduction in intra- and inter-laboratory variability, contributing to standardization and high-throughput laboratory workflows. The algorithm is expected to be useful as an auxiliary tool for reading in environments where specialists are scarce. Moreover, the proposed algorithm is expected to be utilized in further application of AI studies using PEP.

## Supporting information

S1 Fig Examples of protein electrophoresis densitogram and gel images with each of six findings. (A)-(F) is images for acute phase protein, hypoproteinemia, monoclonal gammopathy, nephrotic syndrome, polyclonal gammopathy, and normal, respectively.
(DOCX)

S2 Fig Representative true- and false-positive case gel protein electrophoresis images results from Gradient-weighted Class Activation Mapping (Grad-CAM), obtained using DenseNET-121 classification model. Examples of true positive (A) and false positive (B) cases for indentification of monoclonal gammopathy.
(DOCX)
