# Supplementary material for: Development and validation of a deep learning-based protein electrophoresis classification algorithm
Source: PLoS One. 2022 Aug 24;17(8):e0273284. doi: 10.1371/journal.pone.0273284 (PMC9401151; doi:10.1371/journal.pone.0273284)
Supplement: S1 Fig — (A)-(F) is images for acute phase protein, hypoproteinemia, monoclonal gammopathy, nephrotic syndrome, polyclonal gammopathy, and normal, respectively. (DOCX) [file pone.0273284.s001.docx]

**Supporting information**

**S1 Fig. Examples of protein electrophoresis densitogram and gel images with each of six findings.** (A)-(F) is images for acute phase protein, hypoproteinemia, monoclonal gammopathy, nephrotic syndrome, polyclonal gammopathy, and normal, respectively.

(TIF)

| 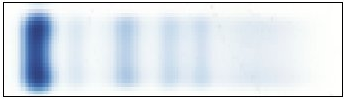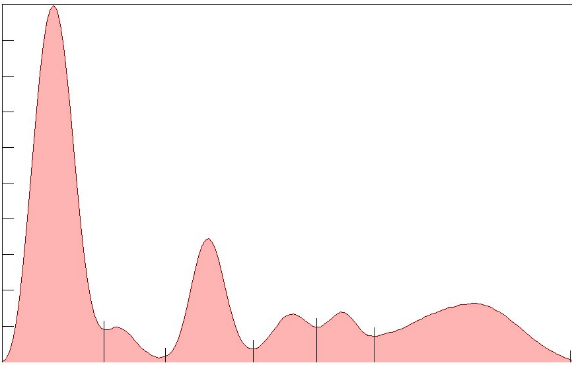Example 1 | 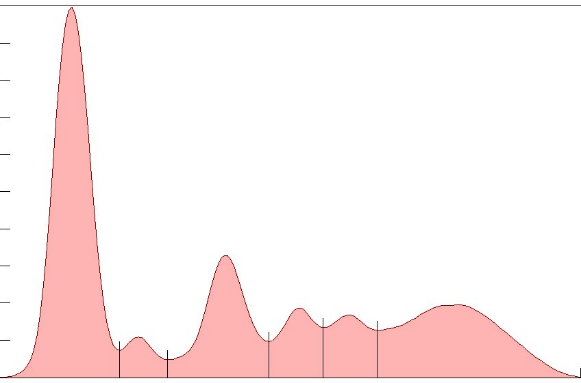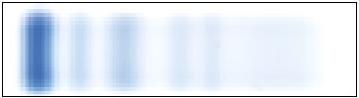Example 2 |
| --- | --- |

(B)

| 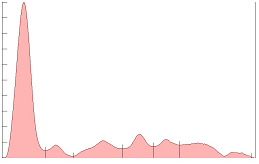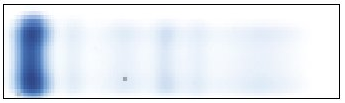Example 1 | 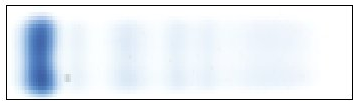Example 2  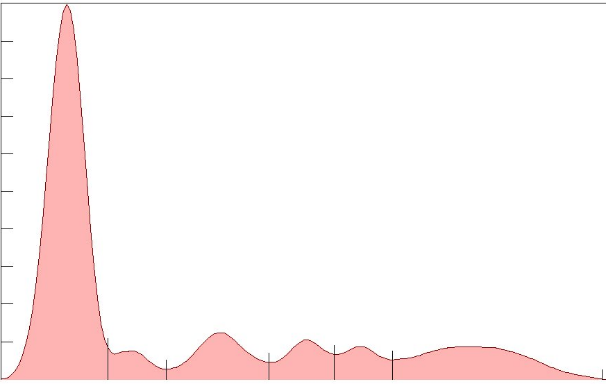 |
| --- | --- |

(C)

| 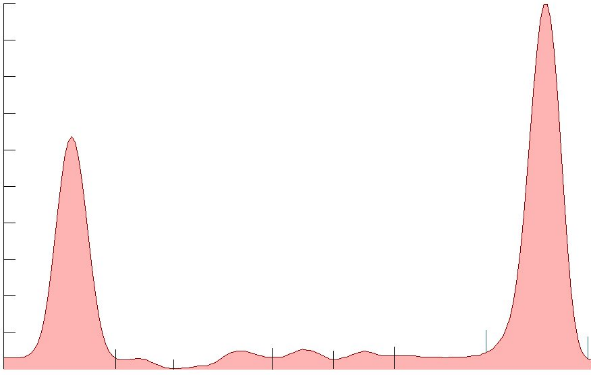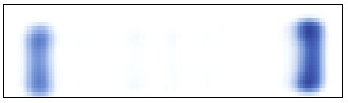Example 1 | 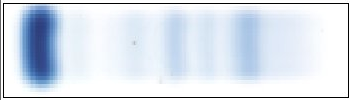Example 2  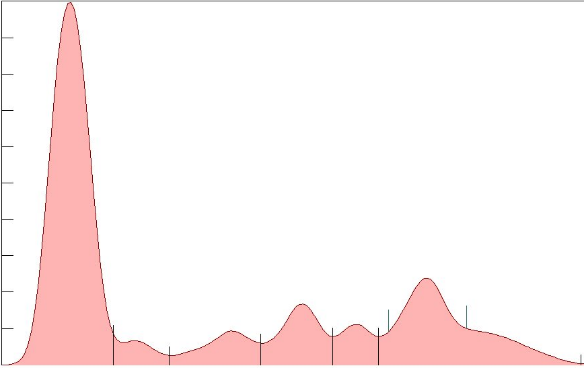 |
| --- | --- |

(D)

| 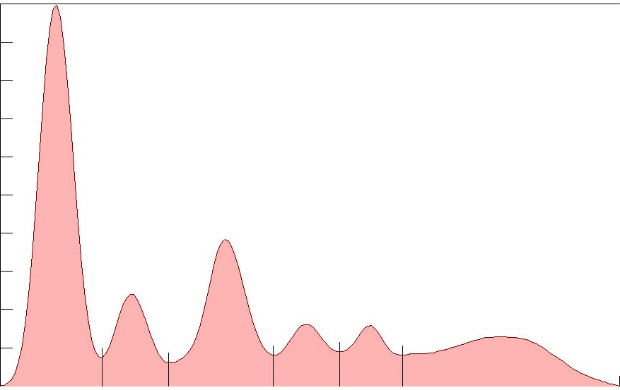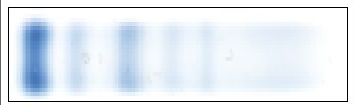Example 1 | 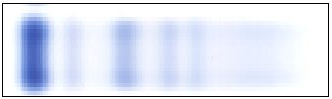Example 2  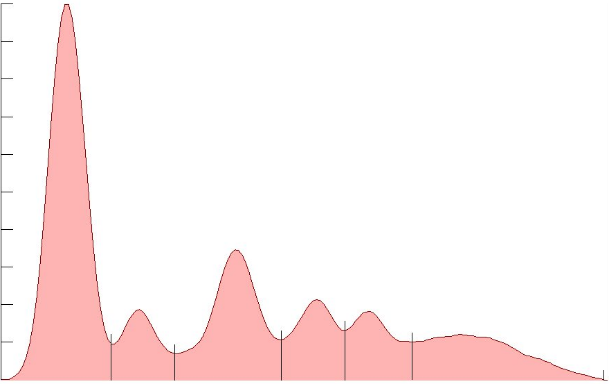 |
| --- | --- |

(E)

| 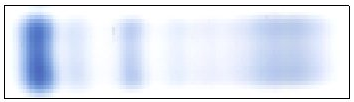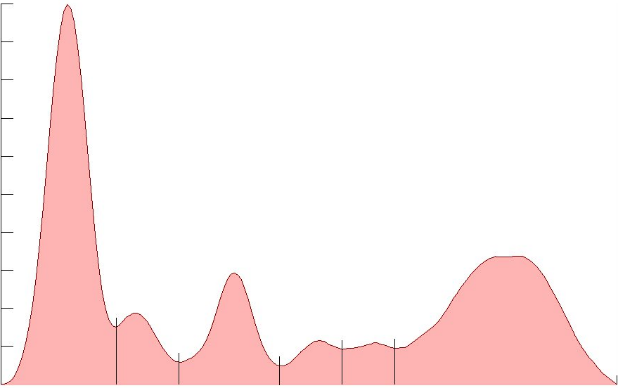Example 1 | 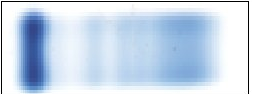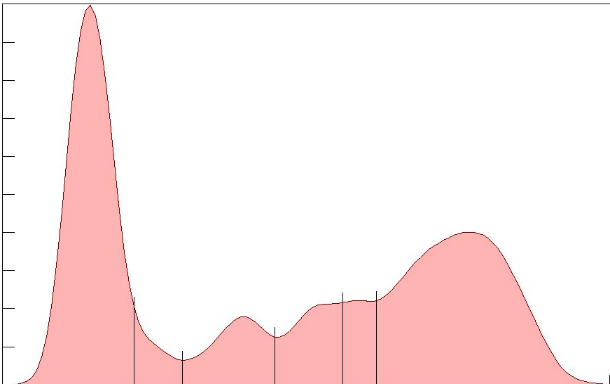Example 2 |
| --- | --- |

(F)

| 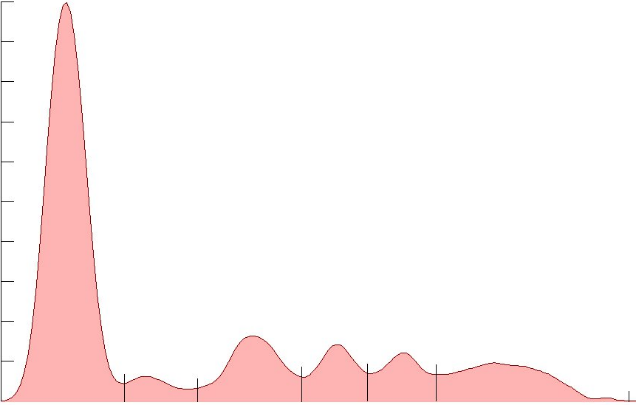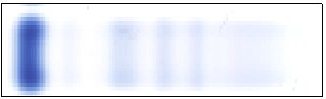Example 1 | 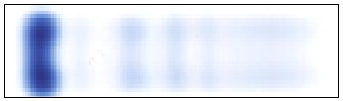Example 2  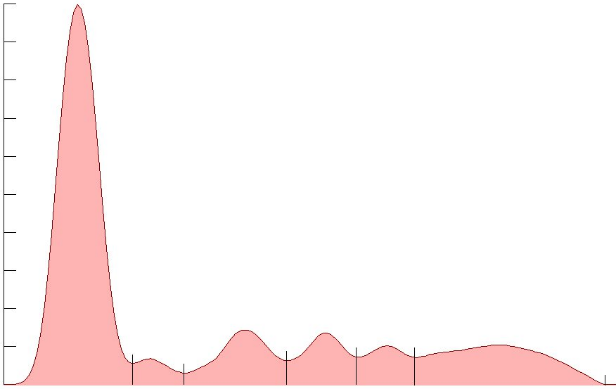 |
| --- | --- |
