# Supplementary material for: Development and validation of a deep learning-based protein electrophoresis classification algorithm
Source: PLoS One. 2022 Aug 24;17(8):e0273284. doi: 10.1371/journal.pone.0273284 (PMC9401151; doi:10.1371/journal.pone.0273284)
Supplement: S2 Fig — Examples of true positive (A) and false positive (B) cases for indentification of monoclonal gammopathy. (DOCX) [file pone.0273284.s002.docx]

**Supporting information**

**S2 Fig. Representative true and false-positive case gel protein electrophoresis images results from Gradient-weighted Class Activation Mapping (Grad-CAM), obtained using DenseNET-121 classification model.** Examples of true positive (A) and false positive (B) cases for indentification of monoclonal gammopathy.

(TIF)


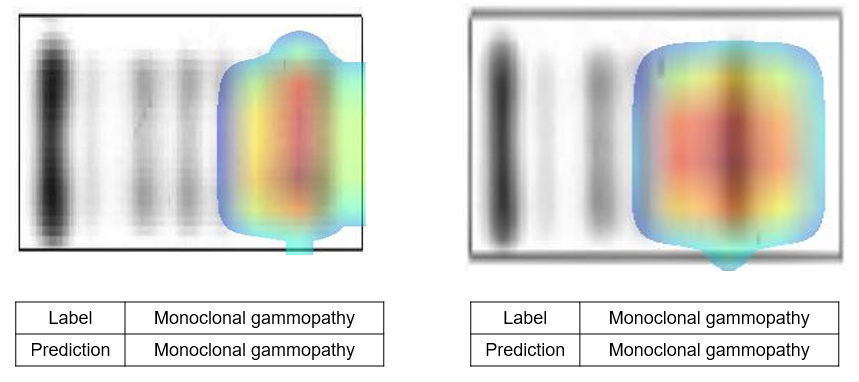
(A)


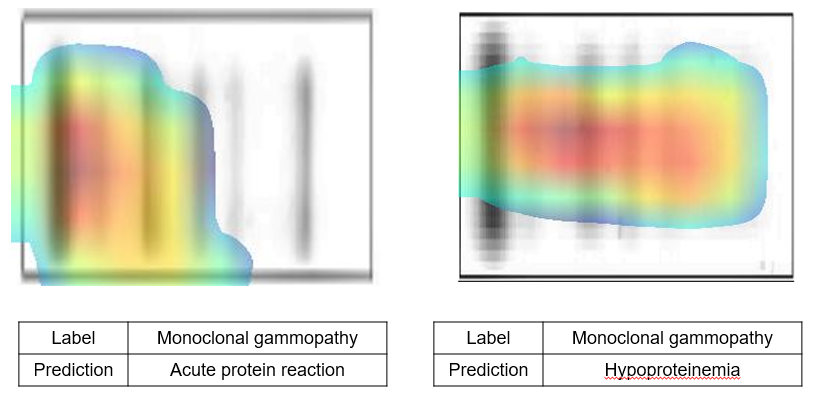

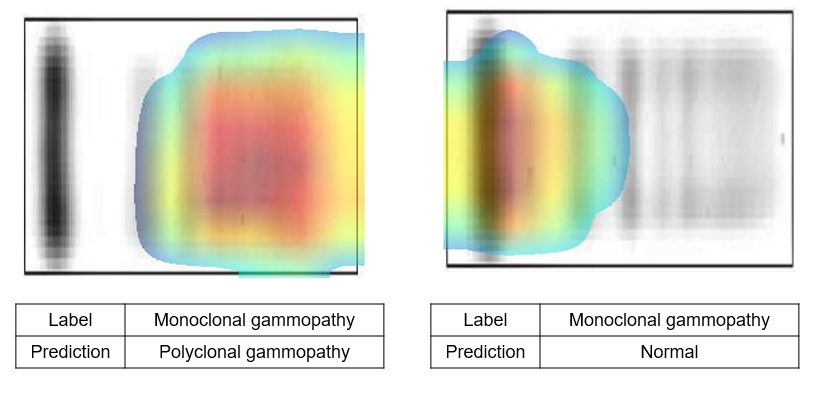
(B)
